# Surgical treatment of cocaine-induced palatal perforations: Report of three cases and literature review

**DOI:** 10.4317/jced.57730

**Published:** 2021-02-01

**Authors:** Javier Barrientos, Guadalupe Corchero, Francisco Soler

**Affiliations:** 1 MD, PhD. Oral and Maxillofacial Surgeon. Maxillofacial Service. Castellón University General Hospital. Avenida de Benicassim 128, 12004, Castellón, Spain; 2 MD. Oral and Maxillofacial Surgeon. Maxillofacial Service. Castellón University General Hospital. Avenida de Benicassim 128, 12004, Castellón, Spain; 3 MD. Oral and Maxillofacial Surgeon. Head of Maxillofacial Service. Castellón University General Hospital. Avenida de Benicassim 128, 12004, Castellón, Spain

## Abstract

Destruction of the osteocartilaginous framework of the nose and sinuses is a well-known side effect of inhaled cocaine. Palate involvement is, however, a very uncommon event that may lead to oronasal communication with the subsequent food and liquids reflux and nasal speech. Given the addictive character of this etiologic agent, the management of cocaine-induced palatal perforations tends to be conservative by means of a prosthetic obturator. In this paper three cases with surgically treated cocaine-induced palatal perforations are reported and a review of the literature is made focusing on the management of this process. Despite the usual reluctancy, surgical palatal reconstruction should be considered in selected committed patients as a definitive solution for the annoying rhinolalia and solid-liquid reflux to the nose, thus improving their quality of life and social relationships.

** Key words:**Cocaine, oral fistula, palate, palatal perforation, oronasal communication.

## Introduction

Cocaine is a crystalline alkaloid obtained by conversion of some subtypes of the coca leaf (Erytroxylum coca and others) into a paste and then into cocaine hydrochloride by means of catalyzing agents like ether, gasoline and sulfuric acid. It has an indirect sympathomimetic-mediated psychoactive action increasing the norepinephrine levels and impairing its reuptake, but it is also able to induce direct vascular smooth muscle contraction (1). Cocaine also blocks the sodium channels and thus interferes with nerve transmission leading to an anesthetic effect. In the 19th century, Köller reported its use as an anesthetic agent in his ophthalmologic medical practice and for decades it has been very popular in ENT nose surgery as an excellent topical anesthetic (2,3).

Cocaine usage in Europe is on the rise and consumption prevalence in some countries like UK and Spain even exceed USA rates in adolescents and young adults. Its widespread abuse, particularly among individuals between 18 to 30 years old, has led to a growing number of patients with secondary effects like nose destruction and, less commonly, palate perforation (4).

Cocaine tissue damage is multifactorial. Given the fact that the most frequently administration route as a recreational drug is nose inhalation or snorting, local adverse effects like mucosal lesions begin on the nasal tract due to vasoconstriction and mechanical irritation caused by the crystals of drug and adulterants inhaled at high speed (5). Vasoconstriction is often aggravated by the use of other topic vasoconstrictors to prevent the rebound hyperemia (2,6). Chronic and compulsive intake leads to perichondrium damage, ischemic necrosis and septum perforation. These osteochondral lesions may even take only 3 weeks to develop in intensive inhalation cases. Destruction progresses centrifugally into the nose lateral wall, sinuses and hard or soft palate leading to oronasal or oroantral communication. These local ischemic and traumatic complications are included in the so-called cocaine-induced midline destructive lesion (CIMDL) and is based in the clinical or radiological detection of at least 2 of these signs: (a) septum perforation, (b) lateral nasal wall destruction of the inferior or middle turbinate in the maxillary or ethmoid sinus destruction and (c) hard palate involvement (2,3,7). Another pathogenic route is the recently reported ANCA-associated vasculitis attributed to levamisole, an anti-helminthic agent with immunomodulatory properties that is used as a common cocaine adulterant (even 70% of its composition) as it potentiates its stimulant effects (8). Moreover, impaired mucociliary transport and decreased humoral and cell-mediated immunity takes place leading to bacterial and fungal local colonization with recurrent nasal infections and chronic wound superinfection. Individual predisposition is also another important factor (2).

Clinical findings in cocaine users include chronic rhinitis, snifﬁng, epistaxis, anosmia and halitosis. Nasal septum perforation is asymptomatic and well tolerated but framework destruction leads to a nasal breakdown with a typical flat-depressed dorsum (saddle nose) appearance with widening of the tip. If oronasal fistula is present, regurgitation of liquids and solids to the nose and rhinolalia will appear, sometimes with dysphagia and oropharyngeal pain. Erosion of Eustachian tubes has also been reported (9) and may be associated with ear pathology.

Whereas nasal septum perforation is a well-known side effect with an incidence of 5 % (10), palatine perforation is one of the most rarely published findings of cocaine abuse; it was first reported in 1991 and since then several case series have been published. No epidemiological characteristics of this disorder relating to patient age or gender have been well established, but some authors report great risk for females due to this gender´s intrinsic susceptibility to develop greater connective, cartilaginous and bone inflammatory impairment (4,10). The incidence of complications associated with cocaine abuse is probably greater than estimated due to this particular patient type, as only severe cases ask for medical advice or treatment (2).

Despite evident background of sustained cocaine abuse, complete exploration with nasal smear, nasoﬁbrolaryngoscopy, craniofacial CT and a thorough blood analysis with creatinine and serological markers must be indicated. A palatal perforation border biopsy with immunohistochemical tests is also mandatory. An accurate diagnosis should exclude local aggressive neoplasms or lymphoproliferative conditions and necrotizing inflammatory, immunological or infective disorders (Wegener’s granulomatosis, penFigo, sarcoidosis, Churg-Strauss syndrome, actinomycosis, mucormycosis, lupus erythematosus, atrophic rhinitis, rhinoscleroma, osteomyelitis, leprosy, leishmaniasis and tertiary syphilis). Idiopathic cases of midline destructive lesion have been attributed to IgG4-related disease, a novel systemic fibro-inflammatory condition characterized by tumorous swelling of affected organs and serum IgG4 elevation (8). In the medical literature, the main concern is to rule out Wegener´s granulomatosis with polyangiitis as the 2 year survival rate of this disease with no treatment is 10% (2) and some histological features of CIMDL resemble those reported for Wegener´s, including the presence of granulomas (1,11). Moreover, the commonly used ANCA test is not always discriminatory between these two diseases; in fact, c-ANCA positive test, which is highly specific (98%) for Wegener´s, has been reported in an unexpectedly large proportion of cocaine-abusing patients with CIMDL and/or levamisole-induced vasculitis. In difficult cases the palatal perforation itself may be a clinical marker pointing out to cocaine as it is a very uncommon finding in Wegener´s (5). Recently, HNE-ANCA (against human neutrophil elastase (HNE)) has been proposed as a useful CIMDL marker to be included in the diagnostic armamentarium (12).

Given the difficulty to achieve a complete and permanent drug dehabituation physicians are often reluctant to treat these perforations surgically and use to recommend a more conservative management.

## Case Report

Case Series

This paper reports 3 patients with cocaine-induced palatal perforations who were submitted in 2019 to the Maxillofacial Service of Castellon General Hospital for surgical treatment.

All of them had a smoking habit and a sustained cocaine abuse history, assured to have abandoned the habit for at least 6 months and were firmly committed to stay away from it. Common clinical findings were nasal speech, liquids and solids passage to the nose, absence of nasal septum and a flat nose appearance. Routine laboratory tests were within normal ranges. They all underwent microbiological tests and perforation border biopsy so as to rule out other disorders as a previous step for considering surgical treatment. All patients had positive nasal cultures for *Staphylococcus aureus*. Biopsies were negative for inflammatory, oncological and immunological diseases and showed unspecific reparative findings with no granulomas, vasculitis nor neoplastic cells. Computed tomography (CT) was routinely made to evaluate the underlying bone.

**Figure 1:**
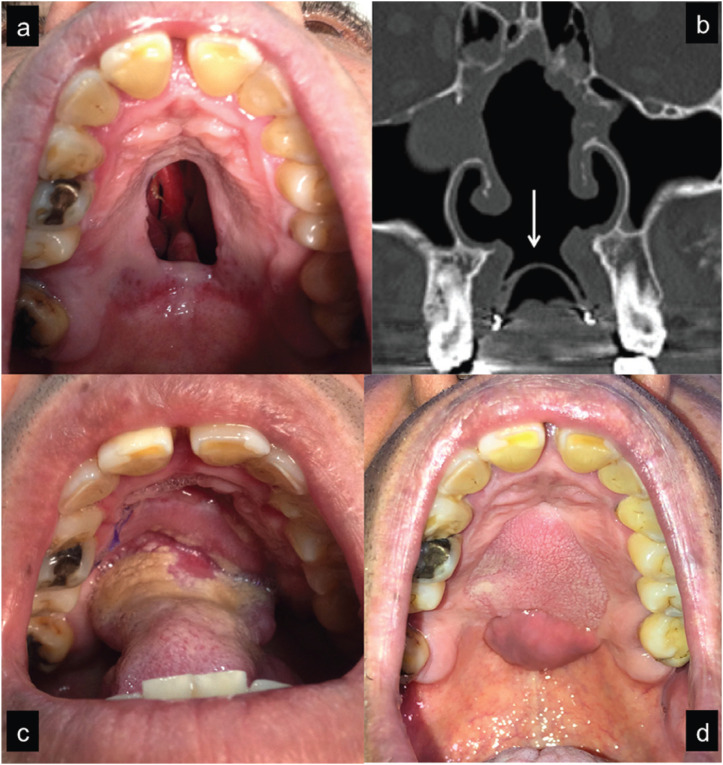
Patient with a 3,5 cm perforation in the hard palate (a). CT scan coronal view (b) showing septum, palate and turbinate bone destruction. Note the presence of an obturator and its sealing effect (mark). Anteriorly based dorsal tongue flap sutured into the defect (c). Postoperative view (d) 6 months after performing a left buccinator flap to cover a fistula that developed in the posterior border of the tongue flap. Note the different mucosa features between both flaps.

The first case (Fig. 1) was a 52-year-old male with a 12 years background of cocaine snorting, reaching up to 2 inhaled grams per day in the late stage when the palatal perforation appeared. He presented a painless ulcer measuring 3,5 cm in the midline of the medium/posterior hard palate that had been conservatively managed with an obturator. Imaging studies revealed a lack of nasal septum and a nasal floor bone defect. Surgical treatment was performed by two layers closure, reconstructing the nasal lining with reverse mucosal flaps from the borders and the oral lining with an anteriorly based dorsal tongue flap. No intermaxillary fixation was made. Unfortunately, in the healing period after the second surgical stage (transecting the pedicle and suturing the posterior border of the flap), extrusion of a necrotic bone fragment was observed and therefore removed, leading to a posterior fistula that was covered after some weeks with a buccinator myomucosal pedicled flap. After six months follow-up the fistula remains covered and the patient reports no air nor liquid leakage to the nose and an improve in his quality of life and social relationships.

**Figure 2:**
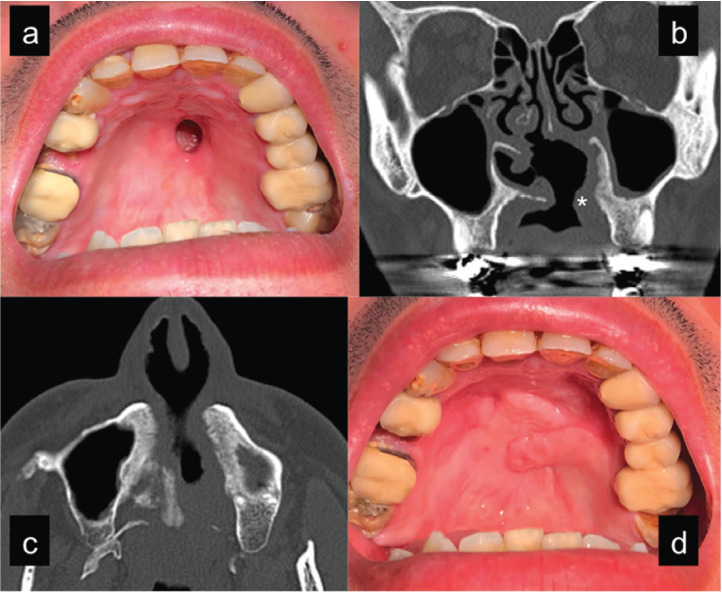
Patient with a 1 cm perforation in the anterior hard palate (a) that can be appreciated (mark) in a coronal CT scan (b). Complete bone loss in the left palatal side in an axial view (c). For this reason the inverted flaps for the nasal lining were partial thickness and the left rotational flap was harvested from the alveolar palatal wall, where underlying bone was present. Postoperative view (6 months) (d) after performing a double opposite pediculated palatal flap.

**Figure 3:**
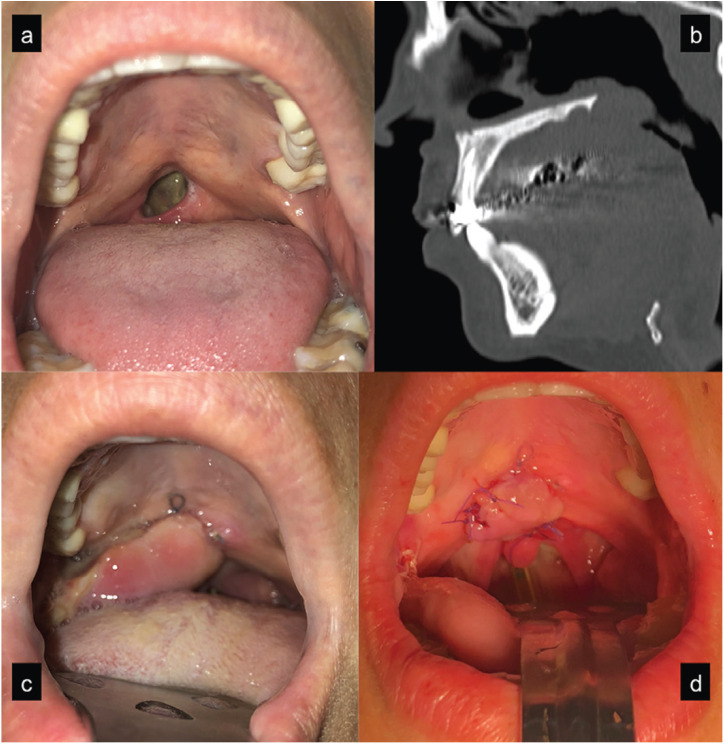
Patient with a 1 cm perforation in the junction between soft and hard palate (a). Palatal bone integrity can be appreciated in the CT scan (b). Posteriorly based miomucosal buccinator flap sutured into the defect (c). Early postoperative view after suturing the transected flap to the posterior border of the defect (d).

The second patient was a 48-year-old man with a history of more than 8 years of cocaine intake reaching up to 4 grams daily in the late phase, when felt some palate swelling and pain for some days that led him to spit a small piece of necrotic bone out. Some days later he noticed a small hole at the anterior-left area of the hard palate when he was not able to make effective negative pressure to smoke a cigarette. The ulcer had a slow growth reaching 1 cm in size, inducing nasal speech and swallowing difficulties due to food reflux to the nose. He enrolled a drug dishabituation program and wore an obturator to seal the defect during that time. At the physical examination point no signs of inflammation or suppuration were found. CT scan revealed septum destruction and a complete lack of bone at the left hard palate, where the communication was located (Fig. 2). Two layers reconstruction was performed using two opposite rotational palatal flaps for the oral side and inverted local flaps for the nasal lining. Care was taken to obtain the left flap from the dentoalveolar area, where underlying bone was present. 6 months later the palatal perforation remains sealed (Fig. 3) and the patient considers that surgical treatment has fulfilled his expectations.

The third case was a 30-year-old female with 1 cm perforation in the soft palate close to its junction with the hard palate. The patient reported one gram per day inhalation of cocaine for a 2-year period. Chronic pharyngitis, halitosis and plugged up ears with recurrent otitis were her main complaints together with the typical swallowing and speech difficulties. However, her main goal was to get rid of the perforation so as to forget her recent addictive past. **Candida* albicans* was isolated in microbiological culture. CT scan revealed septum destruction but complete hard palate bone integrity. Two layers closure was made with inverted local flaps for the nasal lining and a posteriorly based buccinator flap for the oral side. Just some days after the operation the patient referred an objective improvement in speech, though rhinolalia was still present.

## Discussion

Unlike the well-known septum perforation, cocaine-induced palatal perforation is a very uncommon complication. As a result of the increase in cocaine use in the recent years, particularly among young adults, clinicians must consider this condition when making a differential diagnosis.

Some authors reported this process to be more frequent in females because of sex-related predisposition to connective inflammatory disorders (10). Others have suggested that lower blood drug concentrations in females make them increase the intake. In this article, though two males were reported, shorter period and less cocaine intake was necessary for the palatal perforation to overcome in the female patient, which may suggest that this disease is not strictly dose dependent but also influenced by other factors like gender or personal predisposition. What seems obvious is that palatal perforation is the next step in the destructive sequence once the first barrier (septum and turbinates) has fallen (6). As happened with this case series, hard palate is more frequently involved than soft palate or both (7).

The difficulty to achieve a stable-in-time rehabilitation once cocaine intake has become a habit makes specialists reticent to treat these perforations surgically. Treatment should be conservative at the beginning and should be focused in cocaine abuse rehabilitation, which stabilizes the progression of the destructive disease. The patient must be warned about reactivation of the process if new cocaine intake is made and must be instructed in frequent nasal washes to remove crusting and necrotic debris. Sometimes oral antibiotics and / or local debridement of necrotic tissues are necessary due to superinfections over necrotic tissues.

Cocaine-induced palatal perforations are initially dynamic and unstable in size; thus, traditional management is based on a conservative approach by means of a removable palatal obturator. This involves a reliable and well tolerated prosthetic sealing of the defect, which helps to overcome social problems and improves quality of life as it avoids regurgitation of solids and liquids to the nose while swallowing and reduces nasal speech. Indeed, both patients with hard palate perforation in this article were using an obturator and were satisfied with it but claimed for a definitive solution for their problem. It is also the treatment of choice for patients who refuse surgical treatment or aren´t able or do not want to abandon the habit. Besides, it can be relined periodically to improve fitment in unstable-in-time defects. Drawbacks include fitting and retention problems in edentulous patients and loss of sensory feedback from the covered mucosa in case of large obturators resulting in speech, mastication and deglutition impairment (7). Moreover, an obturator can´t be worn 24 hours a day and in some patients is not able to stop liquid and air leakage (6).

As spontaneous healing does not occur, the only permanent solution of these defects is surgical reconstruction. Sometimes this is itself a psychologically motivating goal for the patient to definitively abandon the abuse and to get rid of the cocaine sequelae that act as a reminder of the habit. Only after a reasonable cocaine-free time lapse which confirms patient´s commitment and if defect size remains sTable in time surgical treatment may be considered; otherwise flap failure will overcome and perforation would reappear in the previous margins as cocaine is a vasoconstrictor agent (7). A 6-12 months drug-free period is generally accepted as valid though others even suggest 4 years. Aleatory metabolite urine tests may be recommended to check patient´s accomplishment (8), as cocaine is excreted in urine for 48 to 72 hours following intake. Additionally, margin biopsy has been also proposed (13) to reveal signs of sustained cocaine abuse like vasoactive effects, vasculitis, infarcted areas and polymorphonuclear cell infiltrates.

The aim of this surgery is to seal the defect. The choice of the technique mainly depends on lesion location and size, the residual bone support and soft tissue in the area. Other factors include the daily dose and duration of cocaine addiction, infections, general patient conditions and surgeon´s preferences.

A wide range of techniques have been described. Local flaps are only suitable for small perforations and low palatal vaults. These include direct suture, cleft palate techniques, reverse mucosal flaps or pediculated mucoperiosteal flaps like the palatal flap. The availability of palatal tissue is directly dependent on factors like the underlying bone and perforation size. Thus, local flaps provide a limited amount of tissue given the fact that most of these defects fall into class Ia Okay prosthetic classification (6), which means that they involve hard palate but not the tooth-bearing alveolus. Careful planification based on radiologic findings must be made prior to surgery because osseous destruction uses to be larger than the mucosa perforation and desperiostization when performing the palatal flap may fall into an area with no underlying bone. Besides, bone must be present to let the palatal donor area heal spontaneously. The patient should be warned about the poor predictability of this surgery, as the previous microvascular damage condition of the palate is unknown and these flaps are at risk for partial or complete failure (7,14), especially if they are stretched or sutured under tension. Moreover, reflecting the periosteum may lead to vascular damage of a previously compromised bone. This seemed to be the case in our first patient, as a piece of bone sequestration was extruded and collateral fistula appeared.

Regional flaps like tongue, buccinator, buccal fat pad or temporal muscle flaps may be indicated for small to medium defects. Other pediculated flaps may be obtained from the vomer, nasolabial area, pharyngeal or vestibular mucosa. Regional pedicled flaps are reliable but drawbacks include the possibility of tooth removal and/or blue cement bite block so as to avoid pedicle bite damage if buccinator or buccal fat flap is chosen. On the other hand, tongue flaps may need intermaxillary fixation for 3 weeks to protect the pedicle from stretching. A second surgery is often required to transect the pedicle and to suture the defect area that is covered by the pedicle; this is usually performed under local anesthesia 3 weeks after the first surgical step and may be difficult as vision is unpaired by the mass of the flap, especially if it´s bulky. Flap remodeling in case of bulky flaps should be delayed for at least after 6 months. For medium-to-large defects the temporal flap is a reliable choice as it provides a great volume of well vascularized tissue. However, it is difficult to inset in non-peripheral palatal defects, thus requiring a bone tunnel to be made through the maxillary sinus. Moreover, it fails to achieve a functional rehabilitation in cases of severe soft palate destruction. In this scenario free microvascularized flaps (mainly radial forearm because the pedicle length and flap thickness) may be indicated providing distant and well vascularized tissues (7,14). Interestingly, Di Cossola reports two free-flap-treated patients who restarted cocaine intake, with necrosis resumption at the native palatal mucosa margins surrounding the flap but not in the flap itself, which shows the resistance and reliability of its vascularization. Le Fort I osteotomy with bilateral buccal fat pad flap has been described as an alternative for small-to-medium-sized defects if temporal or free flaps are contraindicated (15).

Nutrition by nasogastric tube is usually recommended during the early healing stage, i.e. 2-3 weeks. Postsurgical clear acrylic-made obturators may be also indicated to protect the reconstructed area while allowing direct inspection of the flaps (6).

## References

[1] Sastry RC, Lee D, Har-El G (1997). Palate perforation from cocaine abuse. Otolaryngol Head Neck Surg.

[2] Smith JC, Kacker A, Anand VK (2002). Midline nasal and hard palate destruction in cocaine abusers and cocaine's role in midline nasal and rhinologic practice. Ear Nose Throat J.

[3] Blanco GF, Madeo MC, Martínez M, Vázquez ME (2017). Case for diagnosis. Palate perforation due to cocaine use. An Bras Dermatol.

[4] Padilla-Rosas M, Jimenez-Santos CI, García-González CL (2006). Palatine perforation induced by cocaine. Med Oral Patol Oral Cir Bucal.

[5] Trimarchi M, Bondi S, Della Torre E, Terreni MR, Bussi M (2017). Palate perforation differentiates cocaine-induced midline destructive lesions from granulomatosis with polyangiitis. Acta Otorhinolaryngol Ital.

[6] Nord GA, Rock A, Murphy FJ, Murphy FJ, Miloslavskiy I, Miller DJ (2012). Prosthetic and surgical management of oronasal communications secondary to cocaine abuse. N Y State Dent J.

[7] Di Cosola M, Turco M, Acero J, Navarro-Vila C, Cortelazzi R (2007). Cocaine-related syndrome and palatal reconstruction: report of a series of cases. Int J Oral Maxillofac Surg.

[8] Berman M, Paran D, Elkayam O (2016). Cocaine-Induced Vasculitis. Rambam Maimonides Med J.

[9] Bacciu A, Ghirelli M, Ingegnoli A, Bozzetti F (2018). Cocaine-Induced Midline Destructive Lesions Associated With Erosion of the Eustachian Tube. JAMA Otolaryngol Head Neck Surg.

[10] Lancaster J, Belloso A, Wilson CA, McCormick M (2000). Rare case of naso-oral fistula with extensive osteocartilaginous necrosis secondary to cocaine abuse: review of otorhinolaryngological presentations in cocaine addicts. J Laryngol Otol Aug.

[11] Silvestre FJ, Perez-Herbera A, Puente-Sandoval A, Bagán JV (2010). Hard palate perforation in cocaine abusers: a systematic review. Clin Oral Investig.

[12] Wiesner O, Russell KA, Lee AS, Jenne DE, Trimarchi M, Gregorini G (2004). Antineutrophil cytoplasmic antibodies reacting with human neutrophil elastase as a diagnostic marker for cocaine-induced midline destructive lesions but not autoimmune vasculitis. Arthritis Rheum.

[13] Silvestre FJ, Salort-Llorca C, Mínguez-Serra MP, Silvestre-Rangil J (2012). Cocaine-related oronasal communication and hard palate destruction. J Investig Clin Dent.

[14] Colletti G, Allevi F, Valassina D, Bertossi D, Biglioli F (2013). Repair of cocaine-related oronasal fistula with forearm radial free flap. J Craniofac Surg.

[15] Pelo S, Gasparini G, Di Petrillo A, Tassiello S, Longobardi G, Boniello R (2008). Le Fort I osteotomy and the use of bilateral bichat bulla adipose flap: an effective new technique for reconstructing oronasal communications due to cocaine abuse. Ann Plast Surg.

